# Supplementary material for: Mesoscale Models for Describing the Formation of Anisotropic Porosity and Strain-Stress Distributions during the Pressing Step in Electroceramics
Source: Materials (Basel). 2022 Oct 1;15(19):6839. doi: 10.3390/ma15196839 (PMC9572883; doi:10.3390/ma15196839)
Supplement: Supplementary file 1 [file materials-15-06839-s001.zip › materials-1899154-supplementary.pdf]

Supplementary material

A. Estimation of the elastic parameters of the matrix outside the soft spherical inclusion

Mixing approaches based on Voronoi structures [25] were employed and analytic formulae for the effective Young's modulus and Poisson's ratios were derived:

$$\frac{E_{eff}}{E_m} = \frac{3(\beta - \alpha)}{(15 + \alpha) + (9 - \beta)\nu_m};\ \bar{\nu} = \frac{(3 - \alpha) + (9 + \beta)\nu_m}{(15 + \alpha) + (9 - \beta)\nu_m} \quad \text{(S1)}$$

where:

$$\begin{cases} \alpha = 2\rho + 2\rho^2 - \rho^3 \\ \beta = 4\rho + 4\rho^2 + \rho^3 \\ \rho = 1 - c \end{cases} \quad \text{(S2)}$$

in which the following notations have been used: E_{eff} is the effective Young's modulus, E_m is the matrix material Young's modulus, $\bar{\nu}$ is the effective Poisson's ratio, ν_m is the matrix Poisson's ratios and c is the average material porosity. On the other hand, an analytical model based on spherical inclusions that estimates both the linear and non-linear mechanical behaviour of composites was developed in ref. [26]. By using this approach in a linear approximation, the effective bulk modulus K_{eff} and effective Lamé coefficient μ_{eff} are expressed as:

$$K_{eff} = K_0 + \frac{c(K_1 - K_0)(3K_0 + 4\mu_0)}{3K_1 + 4\mu_0 - 3c(K_1 - K_0)} \quad \text{(S3)}$$

$$\mu_{eff} = \mu_0 + \frac{5c\mu_0(\mu_1 - \mu_0)(3K_0 + 4\mu_0)}{\mu_0\big((6c + 9)K_0 + 4(3c + 2)\mu_0\big) + 6(1 - c)\mu_1(K_0 + 2\mu_0)}, \quad \text{(S4)}$$

where: K_0, K_1 are the bulk modulus of the matrix and of the inclusion material, respectively, μ_0, μ_1 are the second Lamé coefficients of the matrix material and of the inclusion material, respectively and c is the matrix material's porosity. The equivalence between the quantities predicted by the two theories can be done by using the classical elasticity formulae relating the bulk modulus, Young's modulus, Poisson's ratio and the second Lamé coefficient [*D.K. Singh, Strength of materials, Springer Nature Switzerland AG, 2021*].

In order to correctly apply the analytical model for the material containing a single hollow inclusion, one should determine the bulk modulus and the Poisson's ratios for the considered void. Based on the Hooke's law: $\sigma = E\varepsilon$ where σ is the main longitudinal stress and $\varepsilon = \Delta l/l$ is the specific elongation of the material, it is observed that no matter how much one would like to deform a voided space, the work done to do so is null, meaning that the stress that the material accumulates is null too, *i.e.* $E = 0$. By its definition, the bulk modulus becomes:

$$K = \frac{E}{3(1 - 2\nu)} = 0 \quad \text{(5)}$$

Meanwhile, as the Poisson's ratios defined as the negative of the transversal elongation over the longitudinal elongation, it should be also null, because there is no covariance between the elongations of a voided space whatsoever. Fig. S1, a-b shows comparatively, the calculated values for the effective elastic modulus and Poisson's ratio provided by the two approaches (Voronoi structures [25] and analytical model [25,26]) for variable porosity levels.

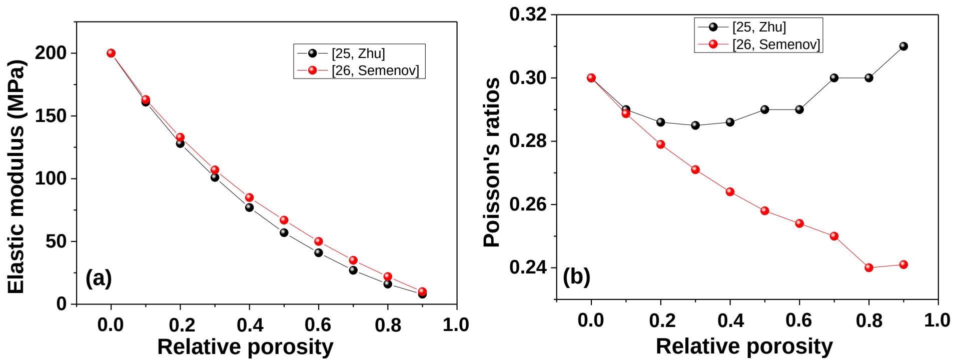

Figure S1. Comparison between the computed elastic moduli (a) and of the Poisson's ratios vs. porosity (b), by using the two approaches.

As observed, there is no significant difference between the elastic moduli (Fig. S1, a), the Poisson's ratios tend to diverge significantly as soon as relative porosity is larger than 20%. Due to this, in the next calculations the values determined in the ref. [26] within the analytical model will be employed, due to the fact that it shows a tendency of reduction when increasing porosity, as it seems natural to be obtained in porous systems.

B. Von Mises stress distribution for a single soft inclusion placed in various radial positions with respect to the cylinder axis

The inclusion was positioned at several radial positions from the cylinder axis and von Mises stress was computed for the case when a single soft inclusion is positioned at several radial positions with respect to the cylinder axis.

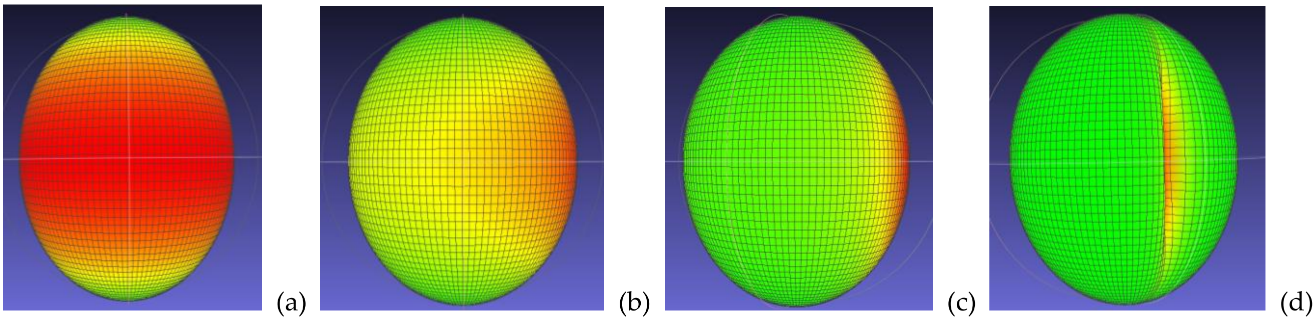

Figure S2. Representation of von Mises stress in a scale colour for the case when a single soft inclusion is positioned at several radial positions with respect to the cylinder axis: $r = 0$ (a), 1.47 mm (b), 2.93 mm (c) and 4.4 mm (d).

C. Comparison of the results obtained for a single soft inclusion by using analytical and numerical approach

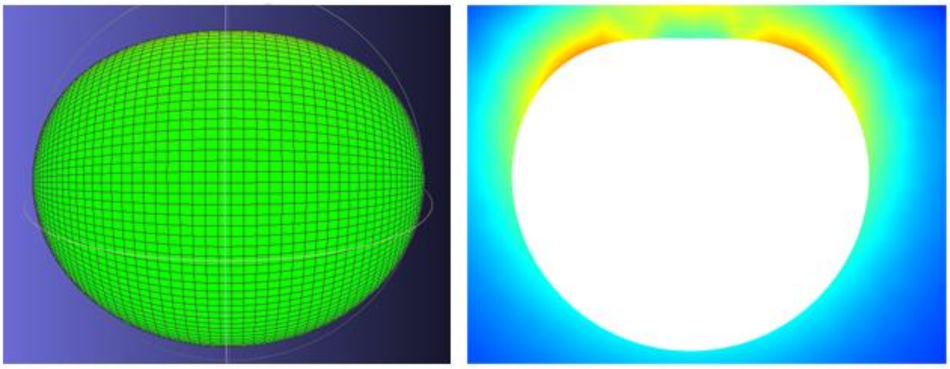

(a) (b)

Figure S3. Qualitative comparison of the results provided by the two models in a longitudinal plane: .

(a) analytical model, (b) numerical model.

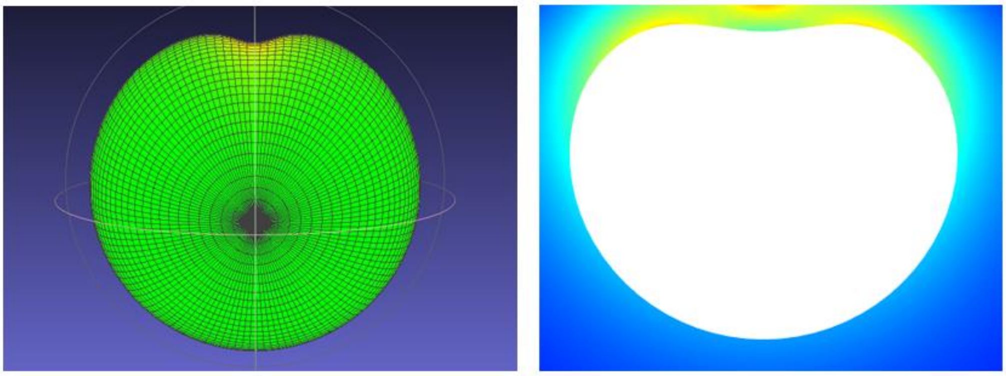

(a) (b)

Figure S4. Qualitative comparison of the results provided by the two models in a transversal plane: (a) analytical model, (b) numerical model.

D. Meshing

As the presented statistical results are obtained after the mediation of the individual results obtained for several structures which are not identical, neither the grid nor the numbers of nodes and elements are not identical. However, as the structures are all randomly distributed, generated using the same governing parameters, the number of elements and the number of nodes sit around 15000 and 10000 respectively. Figure S5 presents some images of the mesh, at different scales.

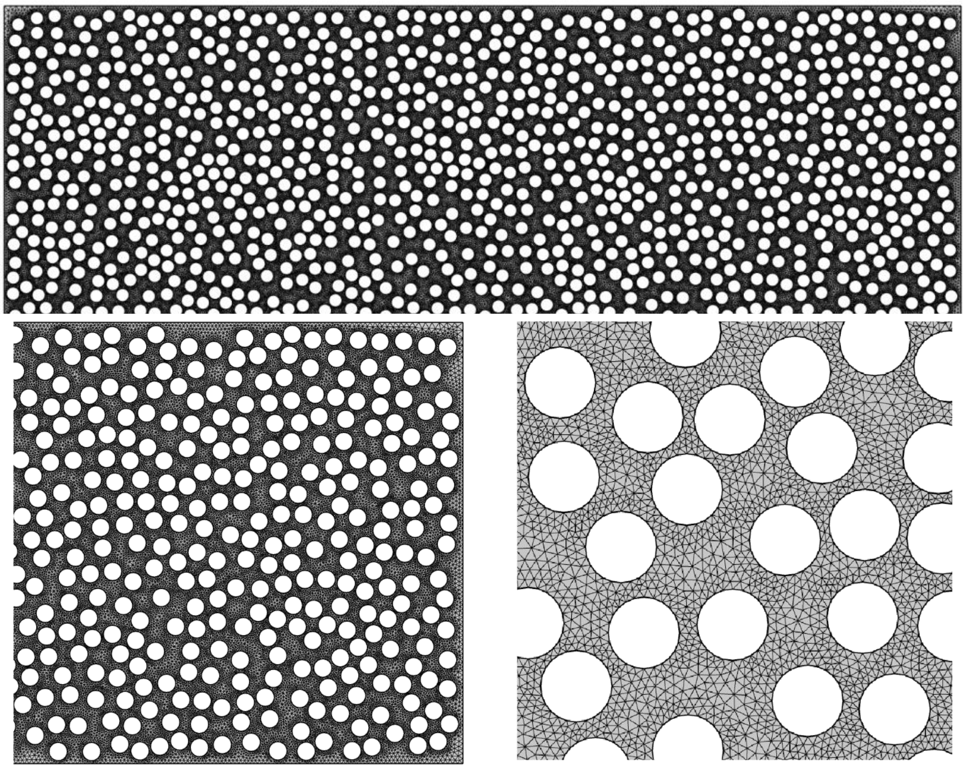

Figure S5. Examples of a meshed system, at different scales.

Figure S6 presents, comparatively, the boundary conditions for the two considered cases.

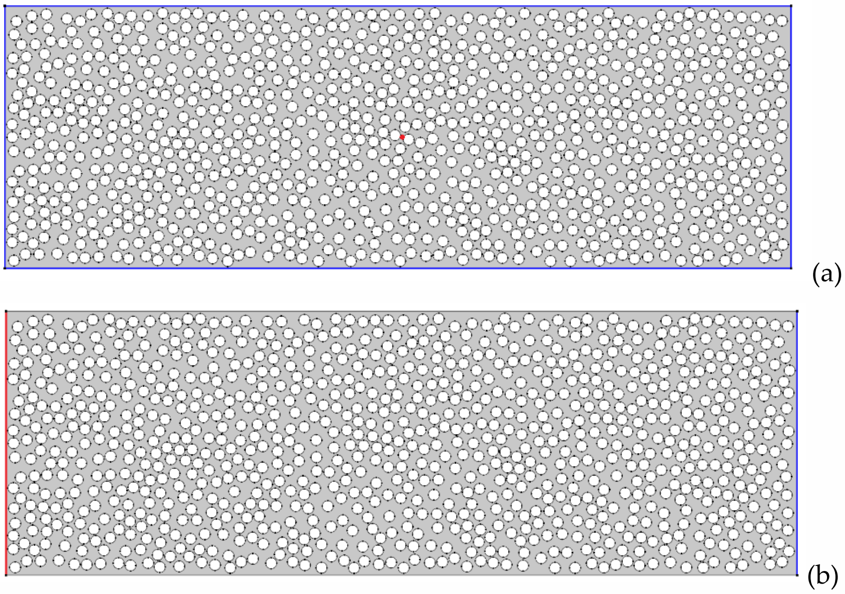

Figure S6. Comparative description of the boundary conditions, in the (a) isostatic case – pressure is applied on the blue, lateral edges, while an anchoring point is defined at the geometrical centre of the structure; (b) uniaxial case – pressure is applied on the blue edge, while the red edge is fixed. All the black edges are free.

E. Determination of anisotropy

The distribution of angles formed by the longest axes of the deformed inclusions with the horizontal cylinder axis was determined as following (Fig. S7, a): (i) an arbitrary point inside the deformed inclusion is chosen and the program draws multiple diverging lines, up to the point when they intersect the frontier of the inclusion; (ii) the shape of the inclusion is approximated to a set of adjacent triangles; (iii) by mediating the position vectors of the mass centre in each triangle, the centre of mass of the shape is approximated. The whole process is repeated several times, up to the point where it converges to a fixed position. After the determination of the mass center, several diameters are once more drawn from that particular point and the longest one is chosen (Fig. S7, b). The calculated angle values are stored in specific files and then represented as statistical charts. When the number of the inclusions increases, the distribution becomes more and more stable and its shape takes a *bi-modal character* with almost symmetrical aspect.

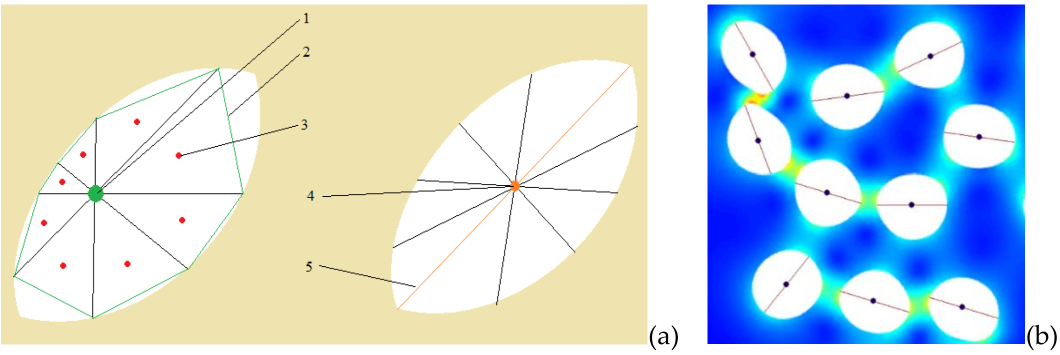

Figure S7. (a) Working steps of the analysis program: (1) arbitrarily chosen initial point; (2) approximated boundary; (3) individual mass center; (4) mediated mass center; (5) longest diameter; (b) Picture generated by the program.

F. Sequential simulations of powder densification process

The linear-elastic model describes only an approximation of the behaviour of such a powder, but, as far as we are concerned, there isn't any simple approach that can be used in such a case. In order to ensure the validity of our approach, we performed a sequence of simulation in which the material properties are modified, at each step, according to the resulting von Mises stress, computed at the previous step. We have chosen, for illustration, a simple, but relevant structure, composed only by two inclusions (Fig. S8,a).

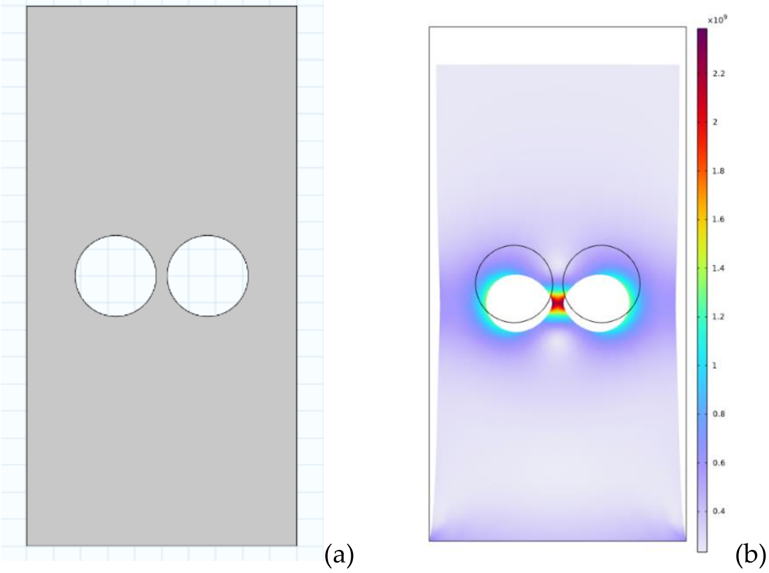

Figure S8. (a) Initial structure; (b) Deformed structure with von Misses stress plot.

As a test case, we performed, firstly, a simulation by using the material properties as described in the manuscript: E = 10^{10} Pa, ν = 0.25, p = 5x10^8 Pa. Figure S8, b shows the deformed structure with von Misses stress plot, in the color scale. We shall consider that, for an average value of the von Misses stress, the material is fully densified. Let us assume that value to be 3x10^8 Pa. In the next steps, the pressure will be gradually increased, and, for each step, the elastic modulus of the material will be locally computed, by using a simple equation, inspired from the Drucker-Prager linear limit, describing, recursively, the dependence of the elastic modulus, against the local von Misses stress:

$$E_n(x,y) = 10^{10} \cdot \frac{\sigma_{n-1}}{3 \cdot 10^8}\ ,\ E_0(x,y) = 10^6\ Pa \quad (6)$$

Scheme 0. p = 5 × 10³ Pa

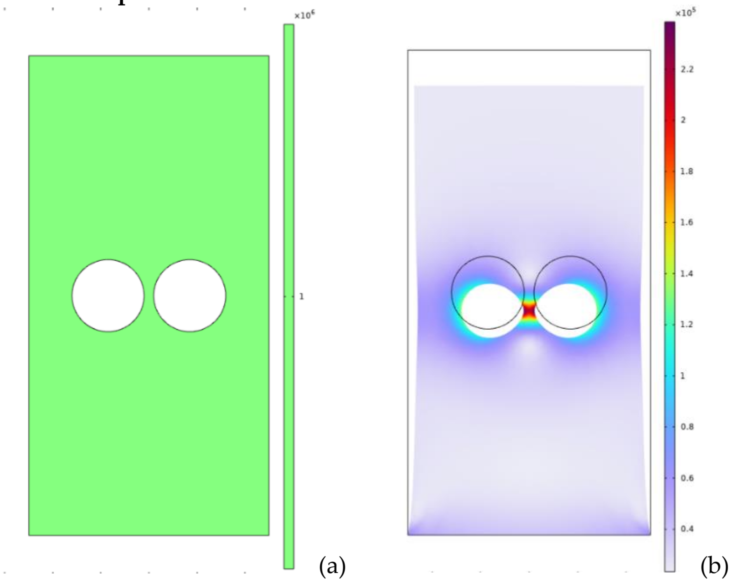

Figure S9. (a) Young's modulus; (b) Deformed structure with von Misses stress plot, at step 0.

Scheme 1. p = 5 × 10⁵ Pa

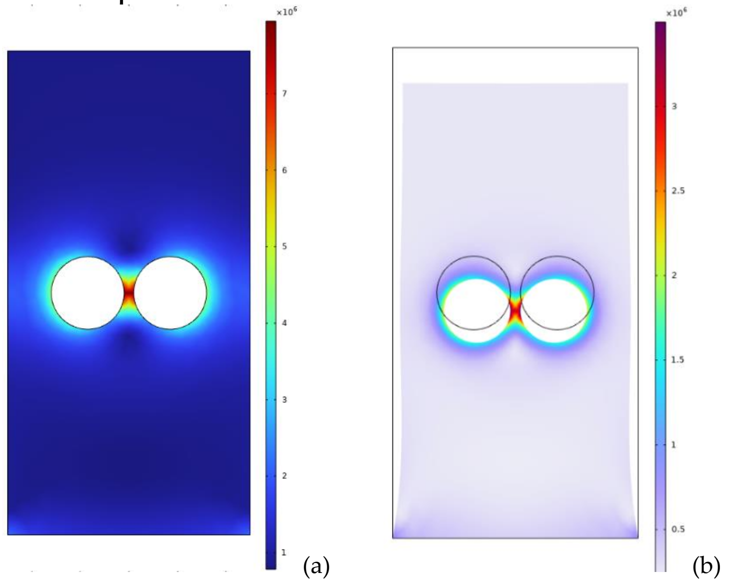

Figure S10. (a) Young's modulus; (b) Deformed structure with von Misses stress plot, at step 1.

Scheme 2. p = 5 × 10⁶ Pa

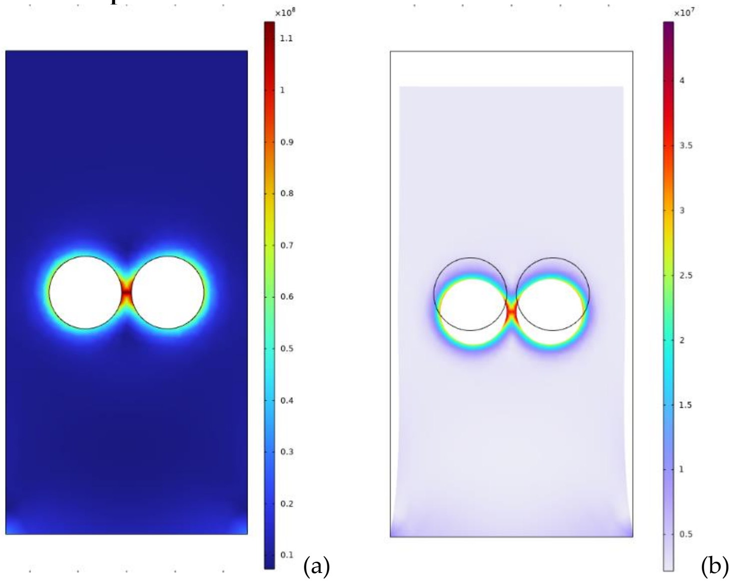

Figure S11. (a) Young's modulus; (b) Deformed structure with von Misses stress plot, at step 2.

Scheme 3. p = 5 × 10⁷ Pa

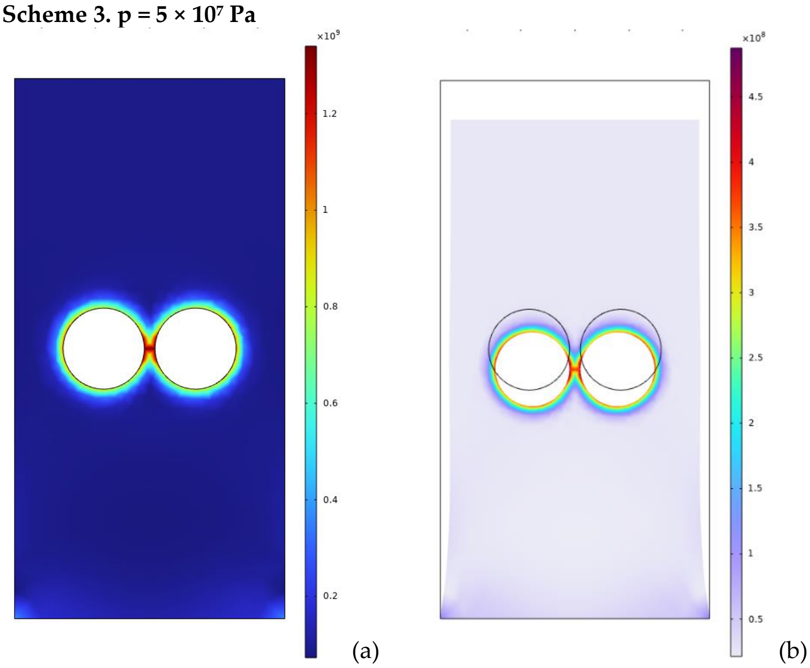

Figure S12. (a) Young's modulus; (b) Deformed structure with von Misses stress plot, at step 3.

Scheme 4. p = 5 × 10^{8} Pa

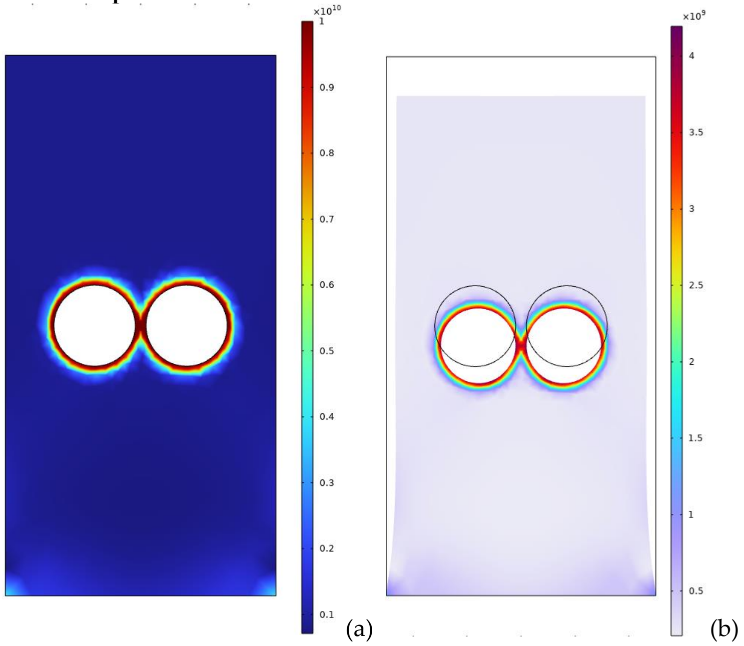

Figure S13. (a) Young's modulus; (b) Deformed structure with von Misses stress plot, at step 4.

Scheme 5. p = 5 × 10^{9} Pa

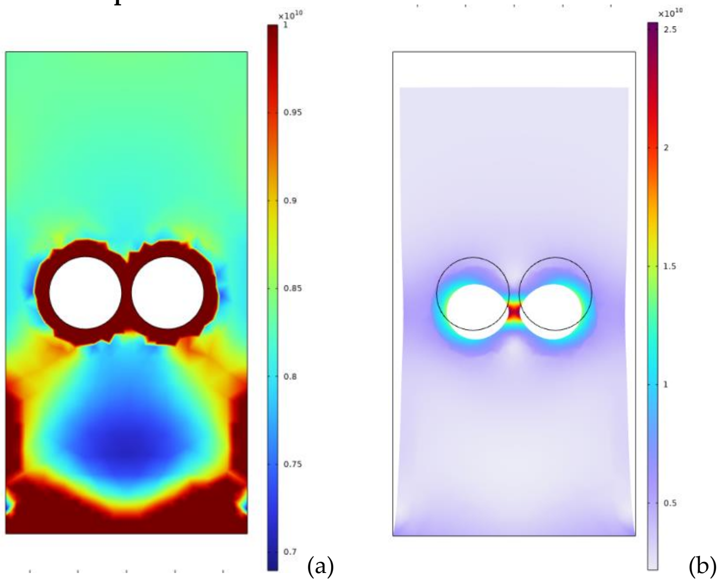

Figure S14. (a) Young's modulus; (b) Deformed structure with von Misses stress plot, at step 5.

Scheme 6. p = 5 × 10^{10} Pa

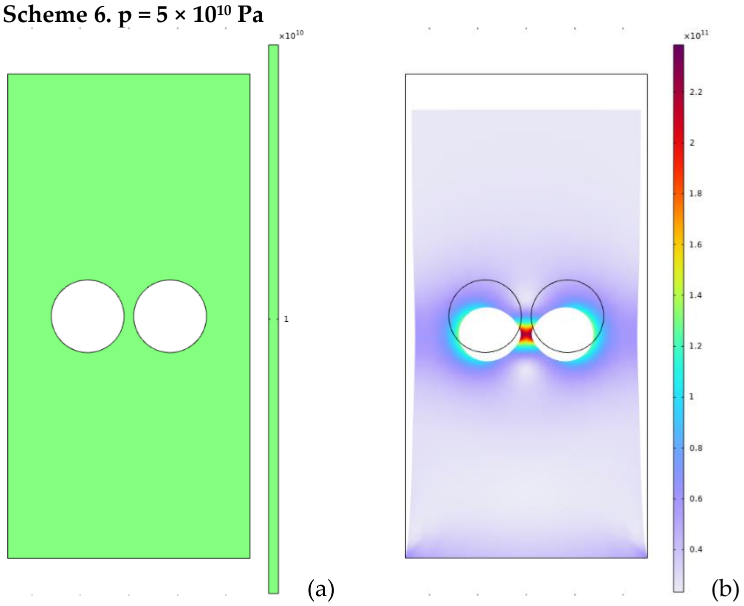

Figure S15. (a) Young's modulus; (b) Deformed structure with von Misses stress plot, at step 6.

Already from step 5 we can distinguish that the shape of the deformed inclusion is similar to the test case, which has been computed with the same input material constants like in the manuscript. So, we can conclude that the present approach is good enough for describing the behaviour of the body during the pressing process.
